# Reproducibility of the brief religious coping inventory with African athletes’ sample using ordinal factor analytical approach

**DOI:** 10.3389/fpsyg.2022.1038202

**Published:** 2023-01-04

**Authors:** Edmond Kwesi Agormedah, Frank Quansah, Medina Srem-Sai, Francis Ankomah, John Elvis Hagan, Thomas Schack

**Affiliations:** ^1^Department of Business and Social Sciences Education, University of Cape Coast, Cape Coast, Ghana; ^2^Department of Educational Foundations, University of Education, Winneba, Ghana; ^3^Department of Health, Physical Education, Recreation and Sports, University of Education, Winneba, Ghana; ^4^Department of Education and Psychology, University of Cape Coast, Cape Coast, Ghana; ^5^Department of Education, SDA College of Education, Asokore-Koforidua, Ghana; ^6^Department of Health, Physical Education and Recreation, University of Cape Coast, Cape Coast, Ghana; ^7^Neurocognition and Action-Biomechanics-Research Group, Faculty of Psychology and Sports Science, Bielefeld University, Bielefeld, Germany

**Keywords:** Africa, athletes, coping mechanisms, factor analysis, religion, reproducibility

## Abstract

**Background:**

Previous studies have revealed that religious coping strategy is common among athletes due to the stressful experiences before and during competitions as part of the mental preparations they go through, the uncertainty of sporting outcomes, and other organizational issues they encounter. This research assessed the reproducibility of the Brief Religious Coping (RCOPE) instrument in an African setting using athletes’ samples from different countries. Particularly, the research sought to assess the (1) factor structure of Brief RCOPE with an African sample, (2) construct validity of the RCOPE measure, and (3) measurement invariance of the RCOPE instrument based on gender and nationality.

**Methods:**

The study surveyed a convenient sample of 300 athletes, including 164 male and 136 female athletes, from 3 African countries (Benin, Ghana, and Nigeria) who participated in the 2018 West African University Games. The Brief RCOPE instrument was administered to the athletes for validation purposes before the competition. Exploratory and confirmatory factor analyses were conducted using the ordinal factor analytic approach.

**Results:**

This validation study confirmed the two-factor dimension (positive and negative religious coping) of the Brief RCOPE measure. Further, all items for each of the dimensions of the inventory contributed significantly to the measure of the Brief RCOPE domains. The positive and negative religious coping dimensions contributed more than half of the variance of their respective indicators. Measurement invariance across gender and nationality was confirmed.

**Conclusion:**

Sufficient evidence was gathered to support the interpretation and use of the Brief RCOPE measure. Coaches and sports psychologists could adopt the Brief RCOPE measure to understand the mental or thought patterns of religious athletes based on existential concerns or stress accrued from impending competitions to inform appropriate religious coping interventions. This notwithstanding, the Minimum Clinical Important Difference (MCID) of the Brief RCOPE should be further investigated to enhance the utility of the instrument for use in intervention-based studies.

## Introduction

Religiosity is an essential part of the existence of several people as a system of convictions related to the presence of a godlike power (Pargament, 2012; Abu-Raiya and Pargament, 2015). Empirical studies have demonstrated that many people rely on religiousness as an internal resource to understand, cope and deal with adverse life events or stressful situations (e.g., Pargament, 2012; Abu-Raiya et al., 2015; Abu-Raiya and Pargament, 2015; Vishkin and Tamir, 2020). For example, persons with professed spiritual backing (e.g., faith in God and use of prayer as coping resources) were more likely to manage better with high levels of life-event stress (e.g., Hagan and Schack, 2017; Hagan et al., 2019; Frimpong et al., 2021; Hagan, 2021; Vishkin, 2021). One mechanism through which religion exerts its positive effects on people during times of stress is religious coping [RC] (Ano and Vasconcelles, 2005; Cummings and Pargament, 2010). According to Pargament (1997), RC is an attempt to understand and cope with life stressors based on sanctities/sacred to gain meaning, control, comfort and closeness to God, or intimacy with others and closeness to God, and/or achieve a life transformation (Pargament et al., 2000, 2011). Like other coping strategies, religious coping can be adaptive or maladaptive (Cummings and Pargament, 2010).

On conceptual and empirical grounds, Pargament et al. (1998) distinguished between two categories of RC: Positive religious coping (PRC) and negative religious coping (NRC). PRC reflects mechanisms that provide a secure relationship with God, a belief that there is a greater meaning to be found, and a sense of spiritual connectedness with others. PRC includes religious forgiveness, seeking spiritual support, collaborative religious coping, spiritual connection, religious purification, and benevolent religious reappraisal (Pargament et al., 2000). Extant researchers have found positive associations between PRC strategies and favorable behavioral outcomes like quality of life, happiness, better physical health, fewer symptoms of psychological distress among individuals (e.g., Pargament et al., 2004; Ironson et al., 2016; Da Silva et al., 2017; García et al., 2017). Conversely, NRC mirrors strategies that represent struggles with one’s relationship with God and/or one’s religious community such as punishing God reappraisals (Pargament et al., 2000, 2011). NRC includes spiritual conflict, spiritual struggle, spiritual guilt (negative self-judgment associated with God), and spiritual anger (frustration expressed towards God; Ironson et al., 2016). Several investigations have linked NRC to unfavorable behavioral outcomes like signs of psychopathology, worse quality of life, lower marital satisfaction, denial and family cohesion, high substance, and higher risk of suicide among different cohorts (e.g., Brelsford et al., 2016; Currier et al., 2017; King et al., 2017; Ng et al., 2017; Parenteau, 2017).

Among inventories developed to measure religious coping (e.g., Centrality of Religiosity Scale [CRS], Huber and Huber, 2021; Duke University Religion Index [DUREL], Koenig and Büssing, 2010); Spanish Brief Religious Coping Scale [S-BRCS], Martinez and Sousa, 2011; Brief Arab Religious Coping Scale [BARCS], Amer et al., 2008; and Iranian Religious Coping Scale [IRCOPE], Aflakseir and Coleman, 2011), it is the Brief RCOPE that has gained much prominence and commonly used religious measurement instrument (Pargament et al., 2000, 2011). The Brief RCOPE is an abridged 14-item version of the full-length 63-item RCOPE scale, representing a different approach to religious assessment grounded in theory and research on coping and religion (Pargament, 1997).

Several studies have shown that the Brief RCOPE has been translated and validated using diverse participants like Christians and Muslims from different jurisdictions such as Europe [e.g., Italy, Poland, Spain, Greece, France, and Portugal] (Paika et al., 2007; Giaquinto et al., 2011; Caporossi et al., 2018; Casaleiro et al., 2022), America (e.g., United States, Brazil, Argentina, Chile, and Puerto Rico; e.g., Mezzadra and Simkin, 2017; Esperandio et al., 2018; García et al., 2021; McGrady et al., 2021; Pagán-Torres et al., 2021) and Asia (e.g., Persia, Pakistan, Iran, and India; e.g., Khan and Watson, 2006; Mohammadzadeh and Najafi, 2016; Grover and Dua, 2019; Rezaeipandari et al., 2021). These validation studies have approved and reported sound psychometric properties of the two-factor structure of the Brief RCOPE. For example, in Europe, Casaleiro et al. (2022) in Portugal and Caporossi et al. (2018) in France found the two-factor structure of the PRC and NRC to be adequate, with similar findings in the United States (McGrady et al., 2021); Chile (García et al., 2021); and Puerto Rico (Pagán-Torres et al., 2021). These investigations endorsed the applicability and reproducibility of the Brief RCOPE among different cohorts. A key observation consistent across these validation studies is that the authors treated the response options for the scale (i.e., “not at all,” “a little bit,” “quite a bit,” “a great deal” or “very often”) as continuous. Although there is a long-standing debate regarding whether such scale options should be treated as continuous or ordinal (Carifio and Perla, 2008), this study attempted to treat the responses as ordinal. The purpose of this decision is not to refute the findings of studies which adopted the RCOPE scale responses as continuous but to expand on the utility of the Brief RCOPE instrument using a different approach and thus, contributing to the validity evidence of the measure. In Sub-Sahara Africa, research on religion and spirituality among different samples has increased in recent years, with some studies in Nigeria (e.g., Amadi et al., 2016a), Uganda (Mutumba et al., 2017), and Ghana (Frimpong et al., 2021; Hagan, 2021). Although these authors employed the Brief RCOPE in their studies, information on the psychometric properties and performance of the scale across the chosen samples were not reported. To date, research on the validation of Brief RCOPE is lacking in Africa. Religiosity is a relevant dimension of Ghanaian culture and many parts of Africa (Hagan et al., 2019). According to Ghana Statistical Service [GSS] (2021), Christians form about 71% of the population, with Muslims constituting 18 percent, 5% of the populace adheres to indigenous or animistic religious beliefs, while 6% belongs to other religious groups or with no religious inclination. Considering that issues connected with spirituality and religion are dominant in Africa, with proof of more doctrinal codes or religious orientation of its people (Hagan and Schack, 2017; Hagan et al., 2019), evaluating the psychometric properties of Brief RCOPE in an African setting to better understand the possible cultural influences that previous studies (e.g., Amadi et al., 2016a; Mutumba et al., 2017; Frimpong et al., 2021; Hagan, 2021; Rashid et al., 2021) have ignored is warranted.

Religion plays a significant function in sports and athletes’ lives spilling over to sporting performance (Hagan, 2021). The relevance of religion in sports is demonstrated by Noh and Shahdan (2020) in their systematic review which found that multiple beneficial roles including performance optimization, improved athletes’ well-being, enhanced confidence, and increased faith in athletes. Indeed, the relationship between religion and sports cannot be underrated. For example, Hagan et al. (2019) demonstrated the relationship between religion and sports by stressing that on the field of play, some players have been found to exhibit spiritual incarnations including using local drinks for pouring libation, and sprinkling objects on the fields. Other reported spiritual-and-religious-based activities openly demonstrated by athletes are praying, going on their knees, raising their fingers toward the heavens, and making the sign of cross (Jirásek, 2015; Hagan and Schack, 2017).

Research has further shown that athletes experience stress before impending competitions due to the mental preparations they go through, the uncertainty of sporting outcomes, and other events which may happen before the competition (Giacobbi et al., 2004; Hoar et al., 2020). Previous studies have found that some athletes adopt religious coping (Sarkar et al., 2015; Frimpong et al., 2021; Hagan, 2021). The adoption of this type of coping approach has been attributed to the long-standing pervasion of spirituality and religiosity embraced by athletes, especially those in Africa, before and during sporting competitions (Dodo et al., 2015; Hagan et al., 2019). Hence, the justification of the re-validation of the Brief RCOPE measure using multi-national athletes in Africa. More explicitly, this research assessed the reproducibility of the Brief RCOPE instrument in West African settings using athletes’ samples from three countries (i.e., Benin, Ghana, and Nigeria). By employing the ordinal factor analytical approach, exploratory and confirmatory factor analyses (EFA and CFA) were performed to understand the latent structure of the Brief RCOPE and as well to examine its construct validity and reliability in the African setting. Particularly, the following objectives guided the research: (1) to examine the factor structure of the Brief RCOPE in the African sample, (2) to assess the construct validity of the Brief RCOPE measure, and (3) to verify the measurement invariance of the Brief RCOPE instrument based on gender and nationality.

Understanding the reproducibility of the Brief RCOPE within the African context provides useful information on the functionality of the instrument in Africa, following the widespread use of the measure (Amadi et al., 2016b; Frimpong et al., 2021). Issues of religion are not distinct from culture and this transcends to religious coping within a particular culture (Croucher et al., 2017; Quansah et al., 2022a). Examining the consistency of the Brief RCOPE measure across different nationalities (cultures) offers insight into the utility of the instrument in scaling athletes into their religious coping activities. Previous research has established significant gender differences in religious coping strategies, with mixed results (Thomas and Barbato, 2020; Francis et al., 2021; Fatima et al., 2022). Given this, it is not well understood whether these variations from previous studies are emanating from the measurement procedure. This study throws more light on the accuracy of the Brief RCOPE instrument across gender.

## Materials and methods

### Participants selection

Three hundred student-athletes from three African countries (i.e., Ghana, Nigeria, and Benin) were conveniently selected to participate in this study using the descriptive cross-sectional survey design. Out of the 300 participants, 100 cases were randomly selected and used for conducting the EFA based on the recommendations of de Winter et al. (2009). The remaining 200 cases were used to perform the CFA guided by Myers et al.’s (2011) assertion that conducting CFA with a sample of 200 is sufficient. These student-athletes were participating in the 2018 West Africa University Games (WAUG) in Nigeria. Half of the total sample were Nigerians (*n =* 150, 50%), followed by Ghanaians (*n =* 96, 32%) and Beninois (*n =* 54, 18%). Male participants were 54.7% (*n =* 164) and females were 45.3% (*n =* 136) with their ages ranging from 19 to 34 years and the 24-year group had the highest number of athletes (*n =* 40). The mean age of the sample was 26 years with a standard deviation of 3.25.

Participants’ competitive status comprised playing either at the regional, national or international levels. The majority of these participants played at the International (*n* = 144, 48%); National (*n* = 125, 41.7%), and Regional (*n* = 31, 10.3%) levels, respectively. Additionally, Christians formed the largest group (*n* = 177, 59%), followed by Muslims (*n* = 87, 29%) whilst participants in other religions like the African Traditional Religion, Buddhism, and Hinduism were the least in number (*n* = 36, 12%). The study participants were students who have been formally admitted into various public universities to study both undergraduate and postgraduate programmes in their home countries at the time of the competition.

To be classified as an international student-athlete, the person must have competed internationally for their home country at varied levels, received national awards and been involved in continental competitions (Hanton et al., 2005). Further, for a national and regional athlete, the individual should have been involved and received specific awards nationally and/or competed and received some awards within a district or a region in their home countries, respectively. Participants took part in five different sporting events involving handball (*n =* 24), basketball (*n =* 24), volleyball (*n =* 24), athletics (*n =* 150) and football (*n =* 78). Team coaches, captains and other delegation leaders were contacted at their places of residence for assistance to recruit the participants at the competition venue.

### Instrumentation

#### Religious coping: Brief RCOPE inventory

Student athletes’ positive and negative religious coping experiences were assessed using the 14-item Brief RCOPE Inventory (Pargament 1999; Pargament et al., 2000, 2011). This inventory was chosen because of its suitability, brevity and extensive usage in mainstream psychology. Additionally, the specific items on the inventory match well with the perceived religious coping experiences of the student-athletes. The student-athletes were required to indicate the extent to which they adopted specific religious means of coping with stress associated with the pending competition (i.e., WAUG). The instrument has two main subscales containing 7 items each for both PRC and NRC on a 4-point Likert-type scale ranging from 0 (“not at all”) to 3 (“a great deal”). PRC assesses religion as a means to “find meaning during a difficult situation and establishing a state of well-being and closeness to God.” Examples of items that measure PRC are; “I looked for a stronger connection with God” and “I am trusting God will be on my side.” The NRC views religion as a “neglect or punishment from God,” for example, “I wondered whether God abandoned me” and “I think the devil made this happen.” Added demographic information on the survey instrument includes gender, age, nationality, competitive status and religion of respondents. Each participant was asked to indicate the extent to which they adopted particular religious coping mechanisms as they entered the competition. Internal consistency coefficients previously reported for both PRC and NRC subscales are 0.92 and 0.81, respectively (Pargament et al., 2011).

### Data collection procedure

The Institutional Review Board (IRB) at Bielefeld University approved this survey procedure following the adherence to all ethical standards of the sixth revision of the Helsinki Declaration. Further approvals were sought from competition organizers and delegation leaders of Nigeria, Ghana, and Benin, who were with the contingents during the WAUG 2018 competition. Written informed consent were obtained from all participants before data collection. Enquiring from team captains and coaches of the various teams, student-athletes were recruited directly after the establishment of rapport during separate briefing sessions. Each participant was assured of anonymity and the freedom to withdraw from the study at any point. Further, participants were informed that every piece of information they provide would be kept confidential and used for only research purposes.

Two research assistants helped to distribute the survey instruments with pencils to the participants to respond to after a thorough debriefing was done to explain every item on the survey instrument to the participants. Answered questionnaires were all retrieved and sealed in brown envelopes at the participants’ hostels by the research assistants before the opening ceremony began. This approach was to avoid disrupting student athletes’ competition-related programs. The duration for answering the survey instruments was approximately 10 minutes for each participant. The questionnaire administration was carried out in the English Language.

### Statistical analyses

The univariate descriptive statistics were first computed using the mean, variance, skewness and kurtosis for individual items. The EFA, through polychoric correlation, was conducted using the FACTOR software (version 12.1). The parallel analysis was based on minimum rank factor analysis (extraction method) used for determining the number of factors (latent structure; Timmerman and Lorenzo-Seva, 2011). According to Timmerman and Lorenzo-Seva (2011), several simulation studies have found the minimum rank factor analysis superior to other extraction methods when using ordinal data. The Promin (Oblique) method was utilized as the rotation method for the EFA because of its high recommendation in the literature (Lorenzo-Seva, 1999). The EFA was performed through bootstrapping approach with 5,000 bootstrap samples. The decision on the number of factors to retain was made based on the parallel analysis (Baglin, 2014).

The CFA was performed in the R-environment with the Lavaan package using the diagonally weighted least square (DWLS) estimation approach. Given the relatively sufficient sample size of this research, the DWLS was considered an appropriate estimator just like other estimators such as weighted least squares-mean and variance (WLSMV) in terms of providing accurate parameter estimates (DiStefano and Morgan, 2014). This study proposed a 2-factor first-order CFA model based on limited empirical evidence of the Brief RCOPE supporting a second-order CFA model. Prior to the CFA main analysis, the inspection of the covariance error matrix revealed no covariance error structure. The factor loadings (> 0.50), Average Variance Extracted (AVE, > 0.50), and thresholds were considered as the cut-off values for interpretation (Kline, 2011; Hair et al., 2014). The ordinal reliability alpha was also computed through the polychoric correlation matrix (Ferrando and Lorenzo-Seva, 2016).

The following model fit indices with their associated cut-off values were used for both the EFA and CFA: Chi-square (non-significant value of *p* is required), comparative fit index (CFI, > 0.90), Tucker-Lewis Index (TLI, > 0.90), Goodness of fit index (GFI, > 0.90), Adjusted goodness of fit index (AGFI, > 0.90), Root mean square error of approximation (RMSEA, < 0.06), and Standardized root mean square residual (SRMR, < 0.08; Hu and Bentler, 1999; Kline, 2011). Other residual indices were explored, namely, weighted root mean square residual (WRMR, < 1.0) and expected mean value of RMSR for an acceptable model (this estimate should be larger than the RMSR value; Kelley, 1935). Moreover, measurement invariance testing was performed by comparing different models of group membership based on the gender and nationalities of the participants. The recommendations of Chen (2007) guided the assessment of the invariance: a change of –0.01 in CFI, ≤ 0.015 in RMSEA, 0.015 in SRMR (residual/scalar invariance) or 0.030 in SRMR (metric invariance). For all the CFA models, modification indices were applied where necessary to improve the fit indices of the models.

## Results

### Univariate descriptive statistics

Table 1 presents the descriptive statistics of the specific proxies that are used to estimate the religious coping variable.

**Table 1:** Mean, variances, skewness and kurtosis.

Items	Mean	Variances	Skewness	Kurtosis
Q1	2.591	0.733	1.901	3.051
Q2	2.475	0.768	1.619	1.567
Q3	1.066	1.391	0.529	1.298
Q4	0.953	1.074	0.668	0.847
Q5	1.297	1.297	0.443	1.233
Q6	0.941	0.941	1.087	0.031
Q7	1.183	0.697	0.697	0.877
Q8	1.282	1.359	0.244	1.424
Q9	1.787	1.277	0.379	1.263
Q10	1.146	1.274	0.391	1.296
Q11	1.056	1.369	0.514	1.311
Q12	1.169	1.290	0.387	1.297
Q13	1.080	1.156	0.404	1.234
Q14	1.585	1.432	0.184	1.493

The mean score for each item ranged between 2.591 (Q1 “*Looked for a stronger connection with God*”) to 0.941 (Q6 “*Tried to put my plans into action together with God*”). The item variances were between 1.391 and 0.733. Whereas the skewness values for the items ranged between 0.184 and 2.069 whereas the kurtosis estimates were between 3.051 and 0.031. The skewness and kurtosis values were within the acceptable range. That is, the skewness values were between –2 to +2 while the kurtosis values ranged from –7 to +7 (Byrne, 2010).

### Factor structure of Brief RCOPE

#### Model fit

The goodness of fit indices for the EFA with 14 items are as follows: *χ*^2^ (64) = 133.163, *p* < 0.001; SRMSR = 0.069, CI (0.062, 0.069); CFI = 0.914, CI (0.894, 0.950); AGFI = 0.947 CI (0.947, 0.963); and GFI = 0.963, CI (0.963, 0.974). Apart from the chi-square statistics which showed a poor fit, the rest of the indices (i.e., SRMSR, CFI, ACFI, and GFI) revealed that the model was acceptable and good. Further, the summary statistics for the residual fit showed an expected mean value of RMSR was 0.077 (Kelly’s criterion; Kelley, 1935) with a confidence interval between 0.0648 to 0.0773. The WRMR value was 0.0698 with a confidence interval of 0.062 to 0.070, which also showed a good model fit.

#### Parallel analysis based on minimum rank factor analysis

The output from the parallel analysis is shown in Table 2.

**Table 2:** Parallel analysis.

Items/Factors	Real data % of variance	Mean of random % of variance	95 percentile of random % of variance	ORION
1	26.1971^*^	14.5517	16.7526	0.764
2	20.2703^*^	13.1436	14.8575	0.793
3	9.8888	11.9266	13.2767	
4	8.9086	10.7763	11.8227	
5	6.8420	9.7393	10.7392	
6	6.4110	8.6497	9.5336	
7	5.0636	7.5818	8.4854	
8	4.8200	6.5410	7.5636	
9	4.3453	5.5694	6.6661	
10	3.1367	4.4851	5.7083	
11	2.1174	3.4478	4.6795	
12	1.1771	2.3263	3.4631	
13	0.8224	1.2614	2.3519	

*Percent of real variance greater than random variance. ORION-overall reliability of fully-informative prior oblique N-EAP scores.

The results from the parallel analysis showed that the first two factors were appropriate for the Brief RCOPE inventory. This was because the real data percent of variance had only two of the factors with their value greater than the mean of random percent of variance (Ferrando and Lorenzo-Seva, 2016). Further, the Overall Reliability of Fully-Informative Prior Oblique N-EAP scores for each of the extracted factors were 0.764 and 0.793.

### Construct validity of the Brief RCOPE

#### Model fit

The model fit indices for the two-factor CFA with 14 items are as follows: *χ*^2^ (76) = 526.086, *p < 0*.001; CFI = 0.936, TLI = 0.919, GFI = 0.929, RMSEA = 0.062, and SRMR = 0.058. The chi-square fit yielded a significant value which reflected a poor model fit. This may be due to the large sample size. Nevertheless, the other indices showed a good model fit indicating that the specified model fit the data.

#### Factor loadings, AVE, and reliability

The details of the output on the 2-factor CFA with 14 items are shown in Table 3.

**Table 3:** Factor Loadings, AVE, Ordinal Alpha and Threshold.

Domains	Items	I	Factor loadings	AVE	Ordinal alpha	Thresholds		
						t_1_ (se)	t_2_(se)	t_3_(se)
Positive coping	Q1	Looked for a stronger connection with God	0.908	0.761	0.891	–1.555(0.115)	−1.192 (0.095)	−0.761(0.081)
	Q2	Sought God’s love and care	0.891			−1.583 (117)	−1.095(0.091)	−0.458(0.075)
	Q9	Tried to see how God might be trying to strengthen me in this situation	0.908			−0.878(0.084)	−0.305(0.074)	0.350(0.074)
	Q12	Focused on religion to stop worrying about my problems	0.869			−0.262(0.073)	0.271(0.073)	0.928(0.085)
	Q5	Sought help from God in letting go of my anger	0.931			−0.866 (0.083)	−0.358(0.074)	0.305(0.074)
	Q6	Tried to put my plans into action together with God	0.766			−1.428(0.107)	−0.806(0.082)	−0.159(0.073)
	Q7	Asked for forgiveness for my sins	0.820			−1.065(0.090)	−0.544(0.077)	0.126(0.073)
Negative coping	Q8	Wondered what I did for God to punish me	0.834	0.558	0.874	−0.350(0.074)	0.159(0.073)	0.795(0.081)
	Q3	Felt punished by God for my lack of devotion	0.669			−0.042(0.073)	0.323(0.074)	0.915(0.085)
	Q10	Questioned God’s love for me	0.817			−0.228(0.073)	0.262(0.073)	0.981(0.087)
	Q11	Wondered whether my religious community had abandoned me	0.649			−0.025(0.072)	0.297(0.074)	0.967(0.086)
	Q4	Wondered whether God had abandoned me	0.550			−0.117(0.073)	0.515(0.076)	1.244(0.097)
	Q13	Decided that an evil power (like the devil) made this happen	0.732			−0.193(0.073)	0.279(0.074)	1.192(0.095)
	Q14	Questioned the power of God	0.916			−0.553(0.077)	−0.193(0.073)	0.515(0.076)

Positive coping ~ ~ negative coping = −0.603, *p* < 0.001; Average variance extracted.

**Figure 1:**
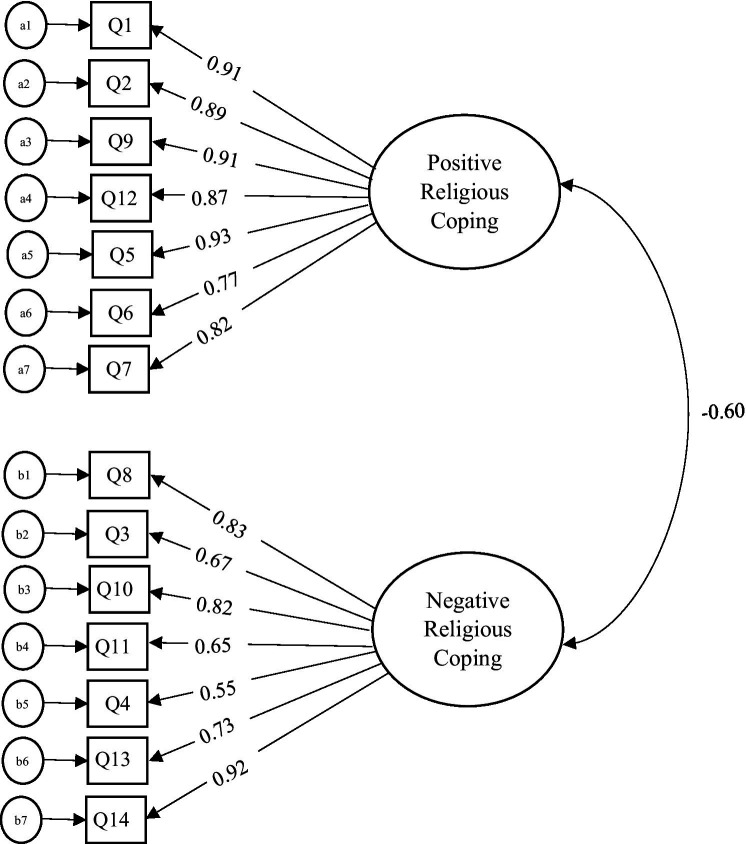
First-order 2-factor confirmatory factor analyses (CFA) model.

The covariance between the factors (i.e., positive coping and negative coping) was −0.603 (*p <* 0.001; see Figure 1). For Factor 1 (i.e., positive coping), the loadings ranged from 0.776 (Q6 “Tried to put my plans into action together with God”) to 0.931 (Q5 “*Sought help from God in letting go of my anger*”; see Table 3) which were found to be sufficient. The AVE value for the positive coping dimension was 0.761 while the reliability estimate was 0.891. The second factor (i.e., negative coping) showed factor loadings for the items between 0.550 (Q4 “*Wondered whether God had abandoned me*”) to 0.916 (Q14 “*Questioned the power of God*”). An AVE value of 0.558 was found for Factor 2 with a reliability estimate of 0. 874 (see Table 3).

The threshold values associated with the specific items showed that the thresholds increased monotonically. For example, item 1 had threshold values of −1.555 (*not at all* vs. *a little bit*), −1.192 (*not at all* vs. *a little bit* and *quite a bit*), and −0.761 (*not at all* vs. *a little bit*, *quite a bit* and *a great deal*). Similarly, item two yielded threshold values of −1.583 (*not at all* vs. *a little bit*), −1.095 (*not at all* vs. *a little bit* and *quite a bit*), and −0.458 (*not at all* vs. *a little bit*, *quite a bit* and *a great deal*). The threshold values depict that participants who were high on the traits were given high scores on the scale.

#### Measurement invariance for gender and nationality

Measurement invariance was tested for gender and nationality to understand whether the RCOPE measures the same construct across the diverse membership of the variables of interest. Table 4 presents the details of the invariance analysis.

**Table 4:** Measurement invariance for gender and nationality.

Indicators	Gender		Nationality		
	Male	Female	Benin	Ghana	Nigeria
Chi-square	1395.76^*^	1443.83^*^	595.39^*^	693.00^*^	1171.41^*^
Degrees of freedom	98	91	91	91	91
CFI	0.963	0.959	0.915	0.932	0.926
TLI	0.946	0.939	0.972	0.981	0.979
GFI	0.939	0.943	0.955	0.961	0.985
RMSEA	0.026	0.021	0.039	0.026	0.028
SRMR	0.038	0.028	0.042	0.045	0.043

* Chi-square test significant at *p* < 0.001.

For gender, separate CFA models were fitted for the male (*χ*^2^ = 1395.76, *p <* 0.001, GFI = 0.939, TLI = 0.946, CFI = 0.963, SRMR = 0.038, RMSEA = 0.026) and female groups (*χ*^2^ = 1443.83, *p < 0*.001, GFI = 0.943, TLI = 0.939, CFI = 0.959, SRMR = 0.028, RMSEA = 0.021). Using the criteria proposed by Chen (2007), gender invariance (scalar, metric, and residual) was confirmed for the RCOPE instrument. Further, different CFA models were also fitted for the various nationalities: Benin (*χ*^2^ = 595.39, *p < 0*.001, GFI = 0.955, TLI = 0.972, CFI = 0.915, SRMR = 0.042, RMSEA = 0.039), Ghana (*χ*^2^ = 693.00, *p < 0*.001, GFI = 0.961, TLI = 0.981, CFI = 0.932, SRMR = 0.045, RMSEA = 0.026) and Nigeria (*χ*^2^ = 1171.41, *p <* 0.001, GFI = 0.985, TLI = 0.939, CFI = 0.926, SRMR = 0.043, RMSEA = 0.028). Inspecting the indicators for each nationality, it was revealed that the measurement invariance was satisfied for nationality for the instrument based on Chen’s (2007) suggestions.

## Discussion

This study assessed the validity (internal structure), reliability, and gender and nationality based measurement invariance of the Brief RCOPE using athletes prior to sports competition (i.e., WAUG) in Africa through the ordinal factor analytic approach. Evident in the EFA, the first two factors accounted for more variations based on the real data than those of the random data, resulting in a two-factor solution of the coping measure for athletes. Additionally, the fit indices for both the EFA and CFA were good, except for the Chi-square test. This was not surprising due to the Chi-square’s susceptibility to large sample sizes (Kline, 2015). The two-factor structure obtained for the Brief RCOPE is consistent with the original version and other studies (Pargament et al., 2000, 2011; García et al., 2021; Rezaeipandari et al., 2021; Casaleiro et al., 2022). These findings support the reproducibility of the Brief RCOPE using athletes’ samples within the African context.

Collectively, all seven items for each of the dimensions of the Brief RCOPE in this study were, at least, internally consistent, and PRC and NRC contributed more than half of the variance of their respective indicators (Hair et al., 2014). This notwithstanding, it is instructive to state that three items from the NRC dimension (i.e., Q3, “Felt punished by God for my lack of devotion”; Q4, “Wondered whether God had abandoned me”; Q11, “Wondered whether my religious community had abandoned me”) had factor loadings below 0.70, and were the least, relatively. These indicators, though fairly good, could not explain up to 50% of the meanings of the respective constructs (Beauducel and Herzberg, 2006). This could be explained from two viewpoints. First, the item structure could account for such findings. For example, a similar sentence structure was observed for the three items (i.e., Q3, Q4, and Q11) except for the terms ‘God’ and ‘my religious community’ used in each case. Although little evidence can be pinpointed to understand this issue, the similarity in the item structure of these items measuring a common factor dimension offers some direction for further investigation. Secondly, the results that the three items with the least factor loadings belonged to the NRC is an indication that, as compared to the PRC, the NRC dimension explains the least variances in the construct. This was reflected in the AVE and the reliability estimates. The trend of results further suggests that the PRC dimension is quite stable and measures a permanent component of religious codes (García et al., 2021). This was further confirmed in a systematic review by Pargament et al. (2011) who found that consistently, the positive coping dimension of the Brief RCOPE showed a high degree of internal structure and reliability across several validation studies.

The AVE values greater than the recommended cut-off of 0.50 suggests the presence of minimal measurement errors which are lower than the explained variances by the domains (positive and negative coping) as well as the precision of the items measuring the construct. This was supported by the relatively high-reliability coefficients confirming that the items measuring each dimension “hang together.” The results on the thresholds suggest that response categories of the Brief RCOPE were appropriately adapted, in that they increased along with the intensity of participants’ use of negative and positive religious coping. This may imply that the participants understood the categories of the responses and were able to distinguish between these categories. Participants who used more positive and negative coping chose responses that matched the intensity of the coping strategies they adopted. Remarkably, three of the items (Q1, Q2, and Q6) functioned best among participants with low PRC. The implication is that, relatively, these items could provide much information on participants’ religious coping strategies, particularly, for those who minimally adopted PRC (Culpepper, 2013).

It emerged that the PRC and NRC strategies inversely covary. This signifies that high PRC is associated with low NRC. In contrast, previous studies have found no relationship (Schanowitz and Nicassio 2006; Ai et al., 2009) and positive relationship (Lewis et al., 2005; Davis et al., 2008) between NRC and PRC. Different cultural settings could potentially explain this discrepancy in the results of these aforementioned studies (conducted in Western societies) and this present study (which was conducted in Africa). Meanwhile, issues of religiosity are highly influenced by culture, such that the way people connect or worship a deity pervades several aspects of their lives (Quansah et al., 2022a). Particularly among athletes, issues of spirituality and religiosity are growing in communities with dominant cultural values like Africa (Hagan, 2021). In line with earlier studies, athletes were found to depend on God in terms of winning trophies, protection against injuries and most importantly, were found to use religiosity in managing and coping with stressors associated with competitions (Damisch et al., 2010; Moore et al., 2013; Dodo et al., 2015; Ofori et al., 2018).

Furthermore, the negative covariance between PRC and NRC measures found in this study can be attributed to the outcomes of adopting each coping measure. For instance, scholars have revealed that individuals who adopt PRC exhibit positive behavior outcomes such as good health, better psychological well-being, enhanced quality of life low level of psychological distress (e.g., Pargament et al., 2004; Ironson et al., 2016; Da Silva et al., 2017; García et al., 2017). NRC, on the other hand, is associated with negative behavioral outcomes such as poor quality of life, and a high level of psychological distress, among others (e.g., Currier et al., 2017; King et al., 2017; Ng et al., 2017; Parenteau, 2017). Essentially, these behavioral outcomes from the two domains of the religious coping measure cannot co-exist; hence, the presence of one automatically reduces the other to the barest minimum. This could explain the existence of a negative association between the two sub-dimensions. Hence, this confirms the ability of the Brief RCOPE instrument to completely scale athletes into those adopting PRC and those utilizing NRC (Cummings and Pargament, 2010).

The measurement invariance hypothesis based on gender and nationality was established with the Brief RCOPE instrument. These findings suggest that the religious coping construct has a similar structure and meaning across male and female athletes as well as athletes from Benin, Ghana and Nigeria. Accordingly, the measurement of religious coping traits can be essentially adopted for use irrespective of the individual’s gender or nationality (Benin, Ghana, and Nigeria). Although previous studies (which adopted RCOPE) have identified gender difference in the utilization of religious coping (Thomas and Barbato, 2020; Francis et al., 2021), the findings from this study provides support to buttress the point that issues of measurement had little to the observed variations in gender. Similarly, the results on invariance in terms of nationality deepen the resemblance of the athletes’ culture across the three nationalities and consequently, their religious codes.

### Limitations

The Brief RCOPE offers no detailed look into other types of religious coping. For example, the NRC of the Brief RCOPE had only divine types of struggle without items related to intrapsychic and/or interpersonal concerns. Hence, future studies could tap into these other dimensions of religious coping to offer a clearer and more detailed understanding of different samples and potential stressors (Pargament et al., 2011). Another limitation is that different types of coping (e.g., problem-emotion focused, avoidance-approach; Quansah et al., 2022b) exist in sports depending on the nature of the sporting event. These other coping forms were beyond the scope of the present study. This validation study should also be interpreted with caution since only the sports athletes’ sample was covered. The outcome of the study may not be applicable to other samples. Therefore, future studies are encouraged to re-validate the Brief RCOPE using different samples in Africa. Despite these limitations, the Brief RCOPE instrument is psychometrically sound and offers a good starting point to assess religious coping in sports across indigenous societies like sub-Saharan Africa where religiosity is a dominant code of its people.

### Practical implications

This study endorses the adoption of the Brief RCOPE instrument for use among athletes in African settings for research purposes. Most importantly, the instrument provides useful grounds for scholars and researchers for scaling athletes into those adopting either NRC or PRC. This scaling of athletes is very necessary because evidence from previous studies has found the PRC dimension to be related to positive behavioral outcomes (e.g., improved quality of life, happiness; e.g., Ironson et al., 2016; Da Silva et al., 2017; García et al., 2017) whereas NRC domain is associated with negative behavioral outcomes (e.g., poor quality of life, depression; e.g., Currier et al., 2017; King et al., 2017; Ng et al., 2017; Parenteau, 2017). With this in mind, coaches and sports psychologists could adopt the Brief RCOPE measure to understand the mental or thought patterns of religious athletes based on existential concerns or stress accrued from impending competitions to inform appropriate religious coping interventions. However, the Minimum Clinical Important Difference (MCID) of the Brief RCOPE should be further investigated to enhance the utility of the instrument for use in intervention-based studies (Li et al., 2019).

## Conclusion

The findings from our validation study confirmed the 2-factor structure of the original Brief RCOPE inventory. Further, a sufficient level of construct validity evidence was gathered in this research to support the utilization of the coping inventory in the context of Africa, specifically, in West African nations. This research starts the discussion on the re-validation of the Brief RCOPE instrument in the African setting using sportsmen and women. The Brief RCOPE instrument is still at its infant stage in terms of its applicability to a non-western sample, supporting the call for more validation studies by the original authors (Pargament et al., 2011).

## Data availability statement

The raw data supporting the conclusions of this article will be made available by the authors, without undue reservation.

## Ethics statement

The Insitutional Review Board (IRB) at Bielefeld University approved this survey procedure following the adherence to all ethical standards of the sixth revision of the Helsinki Declaration. The patients/participants provided their written informed consent to participate in this study.

## Author contributions

FQ and JH conceived the study concept. FQ performed the analysis. EA, FQ, MS-S, FA, JH, and TS prepared the initial draft of the manuscript. All authors contributed to the article and approved the submitted version.

## Funding

The authors sincerely thank Bielefeld University, Germany for providing financial support for the article processing charge of the manuscript through the Open Access Publication Fund.

## Conflict of interest

The authors declare that the research was conducted in the absence of any commercial or financial relationships that could be construed as a potential conflict of interest.

## Publisher’s note

All claims expressed in this article are solely those of the authors and do not necessarily represent those of their affiliated organizations, or those of the publisher, the editors and the reviewers. Any product that may be evaluated in this article, or claim that may be made by its manufacturer, is not guaranteed or endorsed by the publisher.
